# Metabolomics in Diabetes and Diabetic Complications: Insights from Epidemiological Studies

**DOI:** 10.3390/cells10112832

**Published:** 2021-10-21

**Authors:** Qiao Jin, Ronald Ching Wan Ma

**Affiliations:** 1 Department of Medicine and Therapeutics, Prince of Wales Hospital, The Chinese University of Hong Kong, Shatin, New Territories, Hong Kong, China; jinqiao@link.cuhk.edu.hk; 2 Laboratory for Molecular Epidemiology in Diabetes, Li Ka Shing Institute of Health Sciences, The Chinese University of Hong Kong, Hong Kong, China; 3 Hong Kong Institute of Diabetes and Obesity, The Chinese University of Hong Kong, Hong Kong, China; 4 Chinese University of Hong Kong-Shanghai Jiao Tong University Joint Research Centre in Diabetes Genomics and Precision Medicine, The Chinese University of Hong Kong, Hong Kong, China

**Keywords:** biomarkers, cardiovascular disease, chronic kidney disease, metabolomics, type 2 diabetes

## Abstract

The increasing prevalence of diabetes and its complications, such as cardiovascular and kidney disease, remains a huge burden globally. Identification of biomarkers for the screening, diagnosis, and prognosis of diabetes and its complications and better understanding of the molecular pathways involved in the development and progression of diabetes can facilitate individualized prevention and treatment. With the advancement of analytical techniques, metabolomics can identify and quantify multiple biomarkers simultaneously in a high-throughput manner. Providing information on underlying metabolic pathways, metabolomics can further identify mechanisms of diabetes and its progression. The application of metabolomics in epidemiological studies have identified novel biomarkers for type 2 diabetes (T2D) and its complications, such as branched-chain amino acids, metabolites of phenylalanine, metabolites involved in energy metabolism, and lipid metabolism. Metabolomics have also been applied to explore the potential pathways modulated by medications. Investigating diabetes using a systems biology approach by integrating metabolomics with other omics data, such as genetics, transcriptomics, proteomics, and clinical data can present a comprehensive metabolic network and facilitate causal inference. In this regard, metabolomics can deepen the molecular understanding, help identify potential therapeutic targets, and improve the prevention and management of T2D and its complications. The current review focused on metabolomic biomarkers for kidney and cardiovascular disease in T2D identified from epidemiological studies, and will also provide a brief overview on metabolomic investigations for T2D.

## 1. Introduction

Diabetes affected 463 million people in 2019, and it has been projected that 700 million adults will have diabetes worldwide by 2045, with the majority being type 2 diabetes (T2D) [1]. Diabetes is the leading cause of chronic kidney disease (CKD); whereby around 40% of individuals with T2D develop diabetic kidney disease (DKD) [2], and DKD has become the major cause of end-stage kidney disease (ESKD), contributing to half of new cases of ESKD each year [3]. Moreover, individuals with T2D have 2- to 4-fold increased risk of cardiovascular disease (CVD) and death [4]. A multinational study including participants from South and East Asia, North Africa, the Middle East, South America, and Europe reported an approximate 50% prevalence of microvascular complications and 30% prevalence of macrovascular complications in T2D [5]. DKD characterized by impaired glomerular filtration rate or albuminuria has been associated with increased risk of CVD and mortality [6,7]. Furthermore, CVD has been estimated to cause two-thirds of deaths in T2D [8]. Consequently, T2D has been ranked fourth among the disease burden worldwide [9], with a 2- to 3-fold increase in medical expenditures [10].

T2D is believed to arise due to complex interactions between genetic information, developmental exposures and environmental factors such as diet, physical activity, and pollution [11,12]. Hyperglycemia is the hallmark feature of diabetes and has been utilized as a screening and diagnostic biomarker for diabetes, however, metabolic alteration leading to diabetes may be present decades before the onset of hyperglycemia. Modification of lifestyle (diet and physical activity) could delay or even prevent the development of diabetes [13,14], highlighting the utility of powerful screening biomarkers to identify individuals at risk of developing diabetes. Given the increasing risks of adverse outcomes in diabetes and the availability of drugs proven to delay or prevent CVD and DKD [15,16,17], it is also critical to identify prognostic biomarkers involved in the pathogenesis of diabetic complications or predictive of future diabetic complications, which can facilitate clinicians’ decision making and benefit individuals at risk. Biomarkers indicating clinical response to specific medications can help identify individuals who may benefit from the therapy compared with those who have no biological response.

To improve the prevention and risk stratification of diabetes and its complications, as well as to maximize the benefits from interventions, approaches which provide novel insights into the etiology, diagnosis and prognosis of diabetes are much needed. With the rapid advancements in analytical techniques, it has become possible to identify and quantify multiple biomarkers simultaneously in a high-throughput manner, which has dramatically advanced approaches for biomarker discovery.

## 2. Metabolomics and Metabolites

In 1971, Linus Pauling and colleagues introduced the concept of using quantitative and qualitative patterns of metabolites to understand the physiological status within a biological system [18]. Metabolites (with a small molecular mass < 1500 Da) can be endogenous compounds that are produced during endogenous catabolism or anabolism, such as amino acids, peptides, nucleic acids, sugars, lipids, organic acids, and fatty acids (FAs), as well as exogenous chemicals, such as toxins and xenobiotics. The metabolome is termed as the complete collection of metabolites in a given biosample. Metabolomics is the method of comprehensively characterizing the metabolome in cells, organs, biofluids, or other biological systems. Metabolomics is emerging as an attractive tool for biomarker discovery in diabetes and its complications, since metabolites can provide information on the molecular pathways involved in the development and progression of disease.

**Figure 1:**
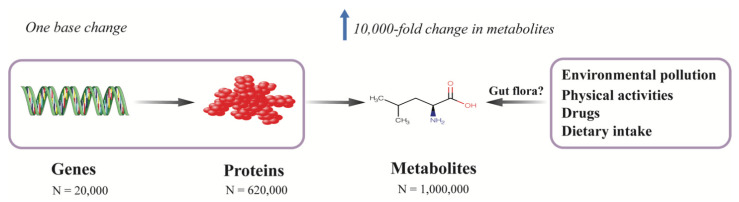
Metabolomics provide a comprehensive molecular profile of a phenotype by integrating both endogenous and exogenous information. Metabolites are the downstream products of the genome, transcriptome, proteome, and enzymatic reactions, which are also affected by environmental exposures, such as environmental pollution, physical activities, medications, and diet. The metabolome is closely correlated with genes in which even one single base change in a protein-coding gene can result in 10,000-fold change in the level of a metabolite. In contrast to the relatively simple chemical constitutions of genome (4 nucleotide bases) and proteome (20 proteogenic amino acids), the metabolome consists of thousands of different chemical classes and the number of metabolites is estimated to be around 1 million, while the number of genes and proteins are about 20,000 and 620,000, respectively. Thus, metabolomics provides a comprehensive molecular profile of a phenotype.

Multiple factors contribute to the development of diabetes. Most genetic variants associated with T2D identified in large genome-wide association studies (GWAS) only modestly contribute to the risk of diabetes. Among the identified genetic variants, over 300 common genetic variants collectively explained only 16% of the variance of diabetes in a study which included around one million individuals of European decent [19]. Metabolites are, in general, the downstream products of gene expression, transcripts, protein transporters, and enzymatic reactions, which are closely correlated with genes in which a single deoxyribonucleic acid (DNA) base change in a given gene can result in 10,000-fold change in the level of endogenous metabolites [20] (Figure 1). Besides internal variations, metabolites can also be affected by exogenous factors, such as diet, lifestyle, physical activities, gut microbiota, and environmental pollution; thus, the metabolome is believed to reflect the molecular profile most proximal to an individual’s phenotype, since it integrates information from the genome, transcriptome, proteome, and enzymes as well as exogenous exposures (Figure 1).

With the advances of analytical techniques and statistical approaches, the number of measurable metabolites has been increasing exponentially over the past 10 years (from 2200 to around 1 million currently) [21]. The application of metabolomics in diabetes and its complications, especially in large-scale epidemiological studies, has facilitated the identification and validation of metabolites that can serve as screening and prognostic biomarkers. Moreover, a multi-omics approach, combining metabolomics with other “omics” data, can provide insights into the complex intercorrelations of different axes involved in the disease and provide opportunities to elucidate the potential causality between biomarkers and disease. The current review focuses on metabolomic biomarkers for kidney and cardiovascular disease in T2D identified from epidemiological studies, and will also provide a brief overview on metabolomic biomarkers for T2D identified in prospective studies. In the following section, we firstly introduce the analytical methods for metabolic profiling.

## 3. Analytical Methods

### 3.1. Untargeted and Targeted Metabolomics

There are two analytical approaches for metabolomics studies: untargeted and targeted. Untargeted metabolomics represents the unbiased approach to complete profiling of the metabolome, aiming to detect, identify, and quantify as many metabolites in a biological sample as possible. The major strength of untargeted metabolomics is the possibility of uncovering novel biomarkers and pathophysiological pathways of disease. However, the annotation of unknown compounds often becomes a challenge, given the wide coverage of signals. In contrast, targeted metabolomics aims to measure a prespecified set or cluster of chemical compounds, which are usually lying on the same metabolic pathways or are structurally similar. Although only capable of providing limited information on the metabolic profiling, targeted metabolomics in general has higher sensitivity and selectivity, and can often provide a deeper understanding of the selected metabolites.

### 3.2. Nuclear Magnetic Resonance (NMR) Spectroscopy

In sharp contrast to the genome, which comprises of only four nucleotide bases, or the proteome, which represents the different combinations of 20 proteogenic amino acids, the metabolome consists of chemical compounds belonging to thousands of different chemical classes [22] (Figure 1). Given the diverse chemical properties and the wide range of concentrations of metabolites, currently, no single platform can quantify the entire metabolome. The two most commonly used techniques are NMR spectroscopy and mass spectrometry (MS), with the latter usually coupled to separation techniques, such as gas chromatography (GC-MS) or liquid chromatography (LC-MS). NMR works by the manipulation of the nuclear spin of certain atomic nuclei, such as ^1^H, ^13^C, ^15^N, and ^31^P, by exposing them to a strong external magnetic field, and then recording the frequency of electromagnetic radiation released as a result of nuclei relaxation. Because the signal of a given nucleus is influenced by the neighboring atoms in a predictable way, the chemical shifts in its resonance frequency can thus be used to identify the underlying molecular structure. Since ^1^H atoms can be found in most of the organic compounds, proton (^1^H) NMR spectroscopy (^1^H NMR) is widely employed in NMR-based metabolomics studies. NMR is noninvasive and nondestructive, and requires little or no sample preparation, chromatographic separation, or chemical derivatization; it can also be applied to in vivo tissues or to living samples, such as the real-time profiling of living cells and analysis of real-time metabolic flux [23,24]. Another advantage of NMR is that NMR is especially suitable for assessing protein-bound metabolites (i.e., lipoprotein particles), and given the high automatability and reproducibility, NMR can be applied in large-scale metabolomics studies [25,26]. The major limitation of NMR, however, is its relatively low sensitivity (millimole to micromole per liter range), which is approximately 10 to 100 times less sensitive than MS.

### 3.3. Mass Spectrometry

The high resolution and sensitivity of MS has made it the most widely used platform for metabolomic studies [27]. Compounds are first separated by chromatographic techniques (i.e., GC or LC) to reduce ion suppression, before quantification and identification by the mass spectrometer. GC-MS is a highly sensitive and suitable method for the separation of volatile and thermally stable metabolites. GC-MS can separate naturally volatile compounds, such as ketones, aldehydes, alcohols, esters, and furans, and compounds that can be made volatile by derivatization, such as sugars, amino acids, lipids, and organic acids [28]. As chemical derivatization may alter the structure of compounds and unpredictable variations may occur during sample preparation, one of the drawbacks of GC-MS is its relatively low reproducibility [29]. Compared with GC-MS, LC-MS requires higher instrument costs, and suffers from lower reproducibility. LC-MS can separate a wide range of classes of compounds, from very polar to very nonpolar ones [30]. As compounds in biofluids must be ionized prior to MS measurement, unlike GC-MS which utilizes the hard-ionization approach (i.e., electron-impact [EI] ionization), LC-MS often uses soft-ionization methods instead (i.e., electrospray ionization [ESI] and atmospheric pressure chemical ionization [APCI]), thus generating ions without fragmentation and allowing the identification of unknown compounds [31,32]. Compared to GC-MS, one of the advantages of LC-MS is that chemical derivatization is not required in most conditions since high temperatures and volatility are no longer required. Moreover, LC-MS is in general more sensitive, covering compounds from low molecular weight to molecular weights beyond 600 Da, including phospholipids, conjugated bile acids, glycosides and sugars [33]. However, the major drawback of LC-MS as an untargeted platform is the lack of transferable mass spectral libraries [34]. Compared with NMR, although MS techniques have higher sensitivity, they are destructive and in general produce results which are comparatively less reproducible. The major advantages and disadvantages of NMR and MS techniques for metabolomic profiling are summarized in Table 1.

## 4. Metabolomics in Diabetes

The characteristic perturbations of metabolic homeostasis associated with or preceding the development of hyperglycemia makes metabolomics a good method to elucidate the potential pathways and to explore potential biomarkers for T2D. Over the past two decades, metabolomics has been widely utilized in large epidemiological studies, and some metabolites/pathways have been identified and validated to be associated with insulin metabolism or being predictive of diabetes across different studies [35]. Table 2 summarizes the findings from some of key prospective studies [36,37,38,39,40,41,42,43,44,45,46,47,48,49,50,51,52,53,54,55,56,57,58,59,60,61,62,63,64,65,66].

### 4.1. Amino Acids

#### 4.1.1. Branched-Chain Amino Acids (BCAAs)

**Figure 2:**
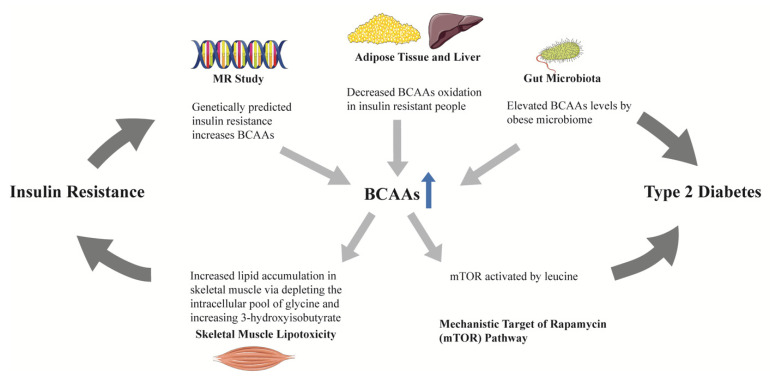
The role of BCAAs in the progression from insulin resistance to type 2 diabetes. In mendelian randomization studies, genetically predicted insulin resistance increased BCAAs, rather than the reverse. BCAAs oxidation in adipose tissue and liver was decreased in people with insulin resistance, leading to elevated circulating BCAAs. Obese microbiomes could elevate BCAAs. One of the BCAAs, leucine, could activate the mTOR pathway. The above findings suggest a potential mediating role of BCAAs in the progression from insulin resistance to type 2 diabetes. Increased BCAAs oxidation in skeletal muscle depletes the intracellular pool of glycine and increases 3-hydroxyisobutyrate production, resulting in skeletal muscle lipotoxicity, which may be the mechanism linking BCAAs and insulin resistance. BCAAs, branched-chain amino acids; MR, mendelian randomization; mTOR, mechanistic target of rapamycin.

BCAAs (isoleucine, leucine, and valine) have been found to be altered among obese vs. lean humans, and were found to contribute to insulin resistance in experimental studies [67]. First reported in the Framingham Heart Study (FHS) and subsequently replicated in the Malmö Diet and Cancer study [38], BCAAs and two aromatic amino acids (AAAs, tyrosine and phenylalanine) were found to be associated with increased risk of T2D during a 12-year follow-up, and the associations remained significant after adjustment for age, sex, body-mass index (BMI), and fasting glucose [38]. The combination of three amino acids (isoleucine, tyrosine, and phenylalanine) could predict T2D (odds ratio [OR] 2.42, 95% confidence interval [CI] 1.66–3.54). Furthermore, compared to individuals from the lowest quartile, people in the highest quartile had an odds ratio of 5.99 (95% CI, 2.34–15.34) for diabetes [38]. Multiple studies across different ethnicities have since replicated the association between BCAAs and risk of diabetes [39,45,47,51,52,53,54,57,59,60]. BCAAs have been found to be related to insulin resistance in animal and human studies [68], however, it remains unclear whether BCAAs contribute to diabetes in a causal manner. Residual confounding and reverse causation in observational studies often hamper the causal inference between biomarkers and outcomes. Using genetic variants mimicking the life-time effects of an environmental exposure which are fixed at conception as the instrumental variable, Mendelian randomization (MR) studies have been utilized to explore the potential causal link between exposures and outcomes. One MR study found that a GRS (genetic risk score) for insulin resistance causally increased BCAAs levels, while genetically increased BCAAs were not associated with insulin resistance [69]. Another two-sample MR study further supported a causal link between insulin resistance and BCAAs [70]. Despite lacking a direct causal link with diabetes, these results suggest that BCAAs may lie on the causal pathway from insulin resistance to diabetes by mediating the effect of insulin resistance on the development of diabetes, since BCAAs levels have been found to be increased by obese microbiomes, and there is decreased oxidation in the adipose tissue and liver in people with insulin resistance [71] (Figure 2). BCAAs may therefore serve as predictive biomarkers, especially given their levels may be increased as early as a decade before overt diabetes.

#### 4.1.2. Aromatic Amino Acids

Tyrosine and phenylalanine, two kinds of AAAs, have also been associated with risk of diabetes [38,39,45,47,49,54,56,59,60]. Analyses in individuals with normal fasting glucose from the FHS found a positive association between phenylalanine and future diabetes, and the consistent findings in MR studies further supported a potential causal role of phenylalanine in the pathogenesis of diabetes [58]. A breakdown product of phenylalanine, 3-(4-hydroxyphenyl) lactate, has been found to be associated with decreased insulin secretion and diabetes in the Metabolic Syndrome in Men (METSIM) study [63]. Results from the Southall Additionally, Brent Revisited (SABRE) study suggested a stronger association of tyrosine with diabetes in South Asians than in Europeans, indicating that the metabolic profile may differ between different ethnicities, and that metabolites may be helpful towards exploring ethnic differences in diabetes incidence. Tyrosine may be an ideal predictive biomarker for diabetes in South Asians [45].

#### 4.1.3. Other Amino Acids

Glycine, a nonessential amino acid [72], was found to be inversely associated with diabetes in Europeans [40,42,47,58], whereas a positive association has been reported in a Chinese population [51]. The MR analysis from the FHS suggested a potential causal link between glycine and diabetes, with the genetically predicted glycine being inversely associated with risk of diabetes [58]. However, a study including 74,124 T2D cases and 824,006 controls did not find an association between genetically predicted glycine and diabetes risk [73]. Furthermore, the study found that genetically higher insulin resistance was associated with lower levels of glycine, suggesting a mediating role of glycine between insulin resistance and diabetes [73]. Alanine, a nonessential amino acid synthesized from pyruvate and BCAAs, has also been reported to be associated with diabetes [39,47,49,54,56]. Glutamate, synthesized from α-ketoglutaric acid in the citric acid cycle, has been found to be associated with the risk of diabetes [47,52,60] and a reverse association of glutamine, a transamination product of glutamate, has been reported [39,52]. The biological roles of these amino acids in the development of diabetes are, however, yet to be elucidated.

### 4.2. Organic Acids

Alpha-hydroxybutyrate, a product of threonine and methionine, upstream of the tricarboxylic acid (TCA) cycle, has been associated with impaired glucose tolerance and diabetes [41,50,52,53,63]. Ketone bodies are an important alternative energy source during fasting, and levels of ketone bodies represent the balance of production (ketogenesis) and utilization (ketolysis). Acetoacetate, converted from free fatty acids (FFAs), has been associated with impaired insulin secretion and diabetes [43].

### 4.3. Lipids

#### 4.3.1. Lipoproteins

Individuals with T2D commonly exhibit dyslipidemia, namely, high levels of triglycerides and small dense LDL particles, low levels of high-density lipoprotein (HDL) cholesterol, and normal or near-normal low-density lipoprotein (LDL) cholesterol levels [74]. NMR has emerged as a novel method to measure lipoprotein particles [75], and has been applied in investigations on lipoproteins and onset of diabetes. In the Insulin Resistance Atherosclerosis Study (IRAS), very-low-density lipoprotein (VLDL) size and small HDL were associated with increased risk of diabetes, independent of triglycerides and HDL cholesterol in prediabetic subjects [36]. In the Women’s Health Study (WHS), both lipoprotein particle size and concentration have been associated with incident diabetes during 13-year follow-up; VLDL size, total/large/small VLDL concentration, and small LDL and HDL were positively associated with diabetes, while large LDL and HDL were inversely associated [37]. Analyses from Finnish populations have also found a positive association for VLDL and a negative association for HDL [46,59]. Recent analyses from the Prevention of Renal and Vascular End-Stage Disease (PREVEND) study with detailed HDL subspecies measurements reported heterogeneous associations between HDL subclasses and incident diabetes: larger HDL size and particles were associated with lower risk of incident diabetes [66].

#### 4.3.2. Fatty Acids

FFAs are produced during hydrolysis of triglycerides [76]. Under the insulin-resistant state, increased lipolysis leads to overproduction of FFAs. In the METSIM study, saturated FAs were associated with increased risk of diabetes, while an inverse association has been found between unsaturated FAs and diabetes [44]. Furthermore, monounsaturated FAs (MUFAs%) were associated with risk of diabetes in a prospective study combining four Finnish cohorts over 8–15 years of follow-up, and polyunsaturated FAs (PUFAs%), mainly n-6 FAs, were associated with decreased risk of diabetes [59]. A two-sample MR study suggested potential causal associations between FAs and fasting glucose, beta cell function, and diabetes [77]. Genetically predicted linoleic acid, the main n-6 PUFAs, has been consistently associated with lower risk of diabetes in a two-sample MR using different genetic variants and analytical approaches [78]. FAs can be derived from dietary triglycerides and phospholipids and dietary counselling has been shown to modify circulating FAs levels [79]. With possible causal links with diabetes, FAs may be emerging as new intervention targets for the prevention of diabetes.

## 5. Metabolomics in Diabetic Kidney Disease

The kidneys are metabolically active organs involved in modulating the circulating levels of metabolites through filtration, reabsorption, secretion, and metabolism (including catabolism and anabolism). Consequently, changes in metabolite concentrations may reflect kidney function, and these changes may even precede the onset or progression of kidney disease, hence providing insights into the development and progression of DKD. Creatinine is one of the commonly applied metabolites that is freely filtered at the glomerulus, and not reabsorbed, with only limited secretion by tubules [80]. Serum creatinine can be used to estimate glomerular filtration rate (eGFR) noninvasively, however, creatinine is significantly increased at more advanced stages of CKD (CKD stage three and onward) and is affected by age, sex, race, and muscle mass. The identification of early markers is warranted given the availability of treatments which can prevent and delay DKD progression. Metabolomic studies have been applied to investigate blood or urine metabolomic biomarkers for DKD and have provided novel insights into the mechanisms leading to DKD and its progression, which make potential therapeutic targets possible. Table 3 summarized metabolomic investigations in DKD [81,82,83,84,85,86,87,88,89,90,91,92,93,94,95,96,97,98,99,100,101,102,103,104,105,106,107].

### 5.1. Amino Acids

#### 5.1.1. Asymmetric Dimethylarginine (ADMA) and Symmetric Dimethylarginine (SDMA)

ADMA (an inhibitor of nitric oxide [NO] syntheses) and SDMA are arginine metabolites formed during enzymatic methylation of arginine residuals. SDMA is a structural isomer of ADMA and is excreted directly by the kidney without any metabolism. A higher serum level of SDMA has been found in people with DKD [82] and SDMA or its ratio to ADMA was predictive of rapid kidney function decline in T2D, independent of baseline eGFR and albuminuria [89,101]. ADMA is metabolized into citrulline and dimethylamine in the kidneys and has been associated with rapid kidney function decline in T2D, possibly due to endothelial dysfunction [101].

#### 5.1.2. Aromatic Amino Acids

**Figure 3:**
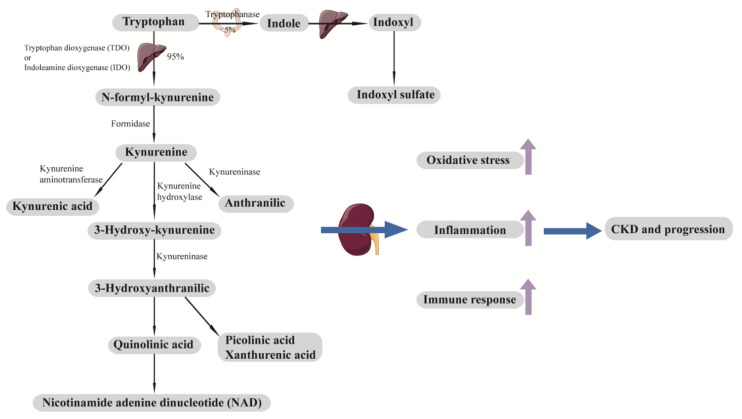
Tryptophan metabolic pathway and development and progression of CKD. Tryptophan is an essential amino acid that cannot be synthesized in the body. A minor fraction of tryptophan (<5%) is metabolized by the indole pathway to produce indoxyl sulfate. Most tryptophan (around 95%) is metabolized by the kynurenine pathway. Downstream metabolites of tryptophan, including indoxyl sulfate, kynurenic acid, picolinic acid, xanthurenic acid, quinolinic acid, and NAD, contribute to oxidative stress, inflammation, and immune response, which lead to the development and progression of CKD. CKD, chronic kidney disease; NAD, nicotinamide adenine dinucleotide.

Both tryptophan (an essential amino acid) and its downstream metabolites, such as kynurenine, are altered in DKD [88,89,91,93,102,103]. Impaired kidney function upregulates tryptophan metabolism pathways and results in increased kynurenine production, stimulating leukocyte activation, cytokine production, oxidative stress, and inflammation [108] (Figure 3). Higher serum levels of tryptophan (or tryptophan/kynurenine) have been found to be inversely associated with rapid eGFR decline in patients with DKD at baseline, independent of baseline renal function [89,102]. Similarly, elevated levels of tryptophan downstream metabolites were positively associated with DKD progression both in patients with type 1 diabetes (T1D) and T2D [88,91,93].

Tyrosine and phenylalanine have also been associated with kidney function and albuminuria in patients with diabetes. A meta-analysis of five cohorts of patients with T2D found a strong inverse association between phenylalanine and baseline eGFR after a comprehensive adjustment for confounding variables, including albuminuria [106], in line with a study comprising three cohorts of patients with T2D [97]. Analyses from the Action in Diabetes and Vascular Disease: Preterax and Diamicron Modified Release Controlled Evaluation (ADVANCE) trial found a crude association of phenylalanine with macrovascular disease and all-cause mortality in T2D, however, adjustment for cardiovascular risk factors attenuated the associations, and phenylalanine was not associated with microvascular disease prospectively [95]. Tyrosine is synthetized by the hydroxylation of phenylalanine through phenylalanine hydroxylase. In the setting of CKD, dysfunctional activity of phenylalanine hydroxylase leads to impaired conversion of phenylalanine to tyrosine, resulting in higher phenylalanine and lower tyrosine [109]. In contrast to phenylalanine, tyrosine has been both cross-sectionally [106] and prospectively [93,95] associated with DKD. A higher level of tyrosine has been associated with higher baseline eGFR [106] and lower risk of microvascular disease in ADVANCE [95]. The downstream metabolite of tyrosine (o-sulfotyrosine) has been positively associated with ESKD in a Joslin proteinuria cohort including patients with T1D, proteinuria and stage three CKD [93].

#### 5.1.3. Other Amino Acids

Leucine and isoleucine have been inversely associated with baseline eGFR in patients with T2D using NMR [106]. However, a study from Steno Diabetes Center Copenhagen using GC-MS found that BCAAs were associated with lower risk of a combined endpoint (kidney dysfunction or all-cause mortality) in patients with T1D [99]. A study from ADVANCE also found that leucine and valine were inversely associated with all-cause mortality in patients with T2D, while alanine, synthesized from BCAAs, was inversely associated with microvascular disease, indicating the complex interactions between BCAAs and diabetes and its complications [95]. Threonine, an essential amino acid involved in the production of glycine, has been associated with lower risk of rapid eGFR decline in patients with T1D [102], and the downstream metabolite of threonine (n-acetylthreonine) was predictive of fast eGFR decline in patients with T2D [91] and ESKD in patients with T1D [93].

#### 5.1.4. Organic Acids Involved in Energy Metabolism

Uracil, a pyrimidine derivative, was altered in urine samples from patients with DKD [85,92]. Pseudouridine, synthesized from uracil, showed association with eGFR decline and urinary albumin–creatinine ratio (UACR) increase in patients with T2D [91] and ESKD in patients with T1D or T2D from studies in Joslin [88,93]. 3-hydroxyisobutyrate, a catabolic intermediate of valine which is produced in mitochondria, has been shown to be altered in patients with DKD [85] and has been found to be associated with ESKD in patients with diabetes in the Chronic Renal Insufficiency Cohort (CRIC) Study [105]. Alpha-hydroxybutyrate, positively associated with insulin resistance and diabetes as mentioned above, however, has been found to be associated with lower risk of ESKD in patients with T2D [88]. Glycine has been found to be reduced in urine samples from patients with established DKD [92], and glycolic acid, an intermediate of glycine, was also reduced [85,92] and was associated with ESKD in analyses from CRIC [105]. Acetoacetate has also been inversely associated with baseline eGFR in patients with T2D [106], and 2-methylacetoacetate, an intermediate of isoleucine metabolism, was reduced in urine from patients with DKD [85]. The abovementioned metabolites are all produced in the mitochondria and are involved in energy metabolism, suggesting that mitochondrial function is dysregulated in DKD.

### 5.2. Lipids

#### 5.2.1. Lipoproteins

HDL particles and their composition (cholesterol and apolipoprotein A1) have been found to be cross-sectionally associated with higher baseline eGFR in studies combining several T2D cohorts using NMR, whereas triglyceride-rich lipoproteins and their lipid components were inversely associated, and HDL particles were also negatively associated with albuminuria [97,106]. A two-sample MR study using the Global Lipids Genetics Consortium (*n* = 188,577) and the CKD Genetics Consortium (*n* = 133,814) suggested a causal link between HDL cholesterol and better kidney function: genetically increased HDL cholesterol was associated with 0.8% higher eGFR and lower risk of incident CKD, and this finding was robust in all the sensitivity analyses; however, there was no strong evidence supporting causal associations of LDL cholesterol and triglycerides with baseline eGFR/UACR or incident CKD [110].

#### 5.2.2. Phospholipids

Phosphatidylcholine (PC) and phosphatidylethanolamine (PE) are the two most abundant phospholipids of mammalian cell types, comprising 40–50% and 15–25% of the total cellular phospholipids, respectively [111]. A case–control study found lower plasma levels of PCs metabolites in patients with T2D and overt DKD (macroalbuminuria or CKD), and this finding was replicated in another group of patients [90]. A prospective analysis from the Cooperative Health Research in the Region of Augsburg (KORA) also found that serum PCs were predictive of incident CKD in hyperglycemic patients, independent of conventional risk factors [104]. Unsaturated PEs have been found to be distinguishable between progressors (≥40% eGFR reduction) and nonprogressors in patients with T2D and baseline eGFR ≥ 90 mL/min/1.73 m^2^ [98]. Sphingolipids are also important constituents of cell membranes and have been involved in cell signaling and activation. Ceramides, the essential precursors of sphingolipids, and sphingomyelin, the most common sphingolipids, were altered in patients with DKD. Higher plasma levels of ceramide metabolites have been reported in patients with DKD [90]; studies from the Diabetes Control and Complications Trial (DCCT) study found that higher plasma levels of very-long-chain ceramides were associated with incident macroalbuminuria in patients with T1D during 14–20 years of follow-up [87]. Sphingomyelin level has been found to be elevated in patients with DKD [84] and was associated with incident CKD in hyperglycemic patients [104] and progression of DKD in patients with T1D [107].

#### 5.2.3. Fatty Acids and Acylcarnitines

Apart from the link between insulin resistance and diabetes, FFAs have also been found to be predictive of DKD progression. Among patients with T2D and baseline eGFR ≥ 90 mL/min/1.73 m^2^, unsaturated FFAs were associated with lower risk of ≥40% eGFR reduction during follow-up [98]. Although associated with macrovascular events and death, FAs were however, not associated with microvascular events or onset or worsening of nephropathy in the ADVANCE trial [112]. Acylcarnitines, involved in the β-oxidation of FAs in the mitochondria and barely detectable in nonpathological conditions, have also been found to be elevated in DKD [90]. C16-acylcarnitine was a strong predictor of fast eGFR decline in patients with T2D and CKD at baseline, independent of traditional risk factors [89]. Disturbed lipid metabolism (remodeling of sphingolipid or impaired β-oxidation of FAs) indicates once again the perturbation of energy metabolism and the role of mitochondrial dysfunction in the development and progression of DKD.

### 5.3. Sodium–Glucose Cotransporter-2 Inhibitors (SGLT2i)

SGLT2i reduced the risk of albuminuria and progression of DKD in patients with T2D in multiple clinical trials [15,113,114], however, its underlying effective pathways remain unclear. Metabolomics have been applied to explore potential molecular mechanisms mediating the protective effects of SGLT2i on DKD. Dapagliflozin has been suggested to improve mitochondrial function. Levels of a panel of urinary metabolites previously linked to mitochondrial dysfunction were increased after 6-week of treatment using GC-MS [115]. A study combining metabolomics (plasma) and transcriptomics (kidney biopsy) found three pathways linked with energy metabolism or mitochondrial function have been affected by dapagliflozin, namely, glycine degradation (mitochondrial function), tricarboxylic acid cycle (TCA cycle) II (energy metabolism), and L-carnitine biosynthesis (energy metabolism) [116]. The improvement of mitochondrial function by SGLT2i as the underlying mechanism to delay the development and progression of DKD further supports the observation that mitochondria play a role in DKD.

### 5.4. Current Challenges in Metabolomics Studies in DKD

The kidney itself can modulate the metabolic pathways, which as a result, affects the levels of circulating metabolites. Furthermore, the definition of CKD in most of the current studies is based on eGFR rather than the measured glomerular filtration rate, while eGFR is insufficient to reflect early kidney dysfunction. Although changes in metabolites may precede the onset or progression of DKD, they may be resulted from early DKD which is not reflected by clinical manifestations or the surrogate markers (i.e., eGFR). For example, tyrosine is positively associated with baseline eGFR [106], as improved kidney function induces increased production of tyrosine from phenylalanine [109]. Tyrosine and its downstream metabolites are also predictive of onset or worsening of nephropathy [95] and ESKD [93], which may be due to the link between tyrosine and kidney function, that tyrosine metabolism as a reflection of kidney function can predict renal outcomes rather than being a physiological pathway. The complex interplay between the kidney and metabolites makes causal inference difficult. However, some metabolites are predictive of DKD independent of baseline eGFR and albuminuria, highlighting their value as prognostic biomarkers. Moreover, the lack of large, prospective cohort studies and independent replications limit the interpretations of these observations and clinical utility of potential biomarkers.

## 6. Metabolomics in Cardiovascular Disease

The heart is responsible for around 10% of the fuel consumption of whole body [117] and beats around 2.5 to 4 billion times over an average human life, even though myocardial energy stores are only enough for several heart beats [118]. To meet these high energy need, the heart consumes more than 20 g of carbohydrates and 30 g of fat per day and uses 35 L of oxygen to generate adenosine triphosphate (ATP) [117]. The metabolism in the heart is highly flexible, such that it can alter the energy utilization rapidly to adapt to the changes in environment via using different kinds of energy substrates, including glucose, fatty acids, ketone bodies, and amino acids [119]. The perturbations of metabolism in the heart can usually be reflected by the changes in the involved circulating metabolites. Detection and quantification of these metabolites provide a way to investigate the underlying pathogenic mechanisms of CVD. Moreover, some of the metabolites have potential to be biomarkers (i.e., screening, diagnostic, or prognostic biomarkers). Metabolomics have been comprehensively applied in studying CVD in the general population and CVD cohorts [119,120]. Table 4 summarizes metabolomics studies in CVD in people with diabetes [95,107,112,121,122,123,124,125,126,127].

### 6.1. Amino Acids

#### 6.1.1. ADMA

ADMA has been found to be elevated in patients with CVD and associated with higher odds of CVD in a cross-sectional study of patients with T2D [122]. ADMA was also predictive of cardiovascular events (CVE) in patients with T2D [124] and patients with T1D and DKD [123]. Higher risks of faster eGFR decline and ESKD in patients with higher ADMA [123] suggest that endothelial dysfunction may be a shared mechanism responsible for vascular complications (cardiorenal complications) in diabetes.

#### 6.1.2. Other Amino Acids

Besides the link with diabetes, BCAAs, tyrosine, and phenylalanine have been found to be associated with intima-media thickness and incident CVD in population-based studies [128,129,130]. Higher phenylalanine was associated with risk of macrovascular outcomes and all-cause mortality after adjustment for age, sex, region, and randomized treatment in the ADVANCE trial, however, further adjustment for other cardiovascular risk factors attenuated the association [95]. Glutamine and histidine, inversely associated with diabetes [39,52], were also inversely associated with macrovascular outcomes in ADVANCE, although adjustment for risk factors attenuated the associations [95]. Although negatively associated with kidney function [106], phenylalanine has been associated with higher risk of incident heart failure and showed added value on the risk-stratification of heart failure [131]. A CVD index composed of six amino acids (ethanolamine, hydroxyproline, glutamic acid, 3-methylhistidine, tyrosine, and tryptophan) was predictive of CVD [125]. The altered levels of amino acids in diabetes, DKD and CVD might suggest some shared pathways or mechanisms leading to diabetes and its complications.

### 6.2. Lipids

#### 6.2.1. HDL

**Figure 4:**
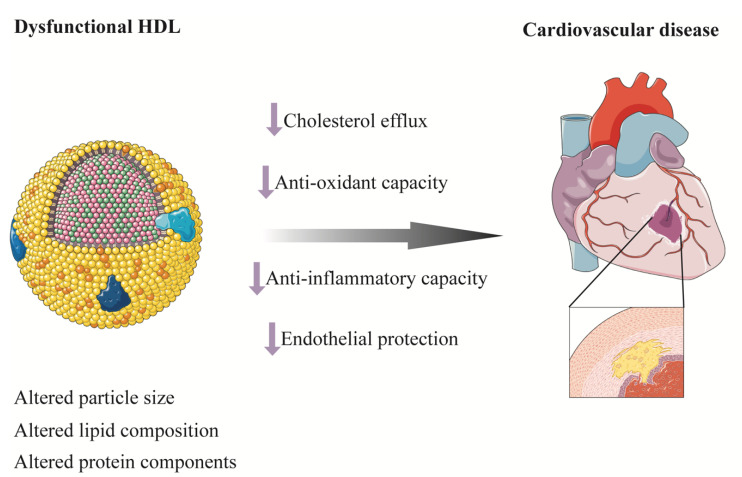
Dysfunctional HDL and cardiovascular disease. HDL are highly heterogeneous in size, structure, composition, and function. Altered lipid composition, protein components, and sizes result in dysfunctional HDL. Decreased cholesterol efflux from macrophages, antioxidant and anti-inflammatory capacity, and endothelial protective function of HDL induce atherosclerosis and cardiovascular disease. HDL, high-density lipoprotein.

Despite the inverse association between HDL cholesterol and risk of CVD in epidemiological studies, MR studies and randomized clinical trials to raise HDL cholesterol level failed to find a protective effect of HDL cholesterol on CVD [132,133,134,135,136,137,138,139]. HDL particles are highly heterogeneous in size, structure, composition, and function [140]. Recent structural and functional studies suggested that the biological function of HDL particles differed in size with small, dense, and protein-rich HDL particles involved in the first step of reverse cholesterol transport (RCT) by mediating the effect of ATP-binding cassette transporter A1 (ABCA1) [141,142]. Besides mediating RCT from macrophages, small HDL particles also have anti-inflammatory, antioxidant, and endothelial protective functions (Figure 4) [143,144,145,146]. In line with this, small HDL particles have been found to be inversely associated with CVD, stroke, CV death, or all-cause mortality in some well-established studies [147,148,149,150,151,152,153,154]. Nevertheless, contrasting findings have also been reported [155,156]. There seems to be a bidirectional relationship between T2D and HDL whereby diabetes could also modulate the composition and function of HDL [157]. Concentration of large HDL particle and HDL particle size have been found to be increased in patients with T1D compared with participants without diabetes, while small HDL and total HDL particle concentration were reduced [158]. A nested case–control study from the Pittsburgh Epidemiology of Diabetes Complications Study found that HDL particle subclasses were predictive of incident coronary artery disease in patients with T1D [121]. Large HDL particle size was associated with risk of death in the Catheterization Genetics (CATHGEN) study and a positive association has been found between higher large HDL particle concentration and death in patients with preserved-ejection-fraction heart failure and patients without heart failure, even after stringent Bonferroni correction and comprehensive adjustment including HDL cholesterol [147]. A nested case–control study from the Prevención con Dieta Mediterránea (PREDIMED) cohort measured HDL functional characteristics and found that lower levels of HDL function markers were associated with higher odds of acute coronary syndrome independent of HDL cholesterol in patients at high CV risk [159]. Taken together, despite a complex interplay between diabetes and HDL, HDL particles or function, rather than simply HDL cholesterol, may be of potential to be prognostic biomarkers and therapeutic targets for CVD (Figure 4). More studies are warranted in this area.

#### 6.2.2. Fatty Acids and Phospholipids

FAs, including n-3 FAs and docosahexaenoic acid, were inversely associated with macrovascular events in a study from ADVANCE, with the associations mainly driven by the associations with CV death and nonfatal stroke [112]. An inverse association between n-3 FAs and death has also been reported [112], indicating the potential of FAs as prognostic biomarkers for CVD in patients with diabetes. Further exploration of the causal role of FAs on CVD may help confirm whether FAs may be therapeutic targets. Apart from the link with progression of DKD, sphingomyelin has been found to be associated with incident coronary heart disease, although further adjustment for CV risk factors attenuated the association [107].

## 7. Intercorrelation of Metabolomic Biomarkers: Limited Predictive Value

Although independent of traditional risk factors, the selected biomarkers usually provided limited predictive value when added over models comprised of conventional risk factors or established risk equations [59,130,131,160]. The highest quantile of a weighted multimetabolite score (0.320 × phenylalanine—0.474 × non-esterified cholesterol in large HDL-0.321 × ratio of cholesteryl esters to total lipids in large VLDL) could predict incident T2D during 15-year follow-up (OR 5.80 [2.22, 15.1]) compared with the lowest quantile, after adjusting for risk factors including BMI, fasting glucose, triglycerides, HDL cholesterol, and HOMA-IR [59]. Addition of the metabolite score over a model including the above-mentioned predictors improved the discrimination and reclassification, with significantly improved integrated discrimination improvement (IDI) and continuous net reclassification improvement (NRI), though the increase in c-statistic was modest and not significant (0.012, *p* = 0.13) [59]. Despite being predictive of adverse outcomes in patients with diabetes, most of the metabolites (sphingomyelin, amino acids and FAs) failed to increase the c-statistic on top of established risk factors [95,107,112]. As demonstrated by Wang and colleagues, the key determinant of the predictive value of multiple biomarkers was the degree of correlation between biomarkers [161,162]. To improve the c-statistic by 0.05, more than 50 moderately correlated (r = 0.4) biomarkers were needed; while when the biomarkers were weakly correlated (r = 0.05), less than 10 biomarkers would be needed to increase the c-statistic by 0.05. Metabolites identified may be enriched in well-recognized pathways associated with diabetes and its complications (DKD and CVD), such as insulin resistance, energy metabolism, cholesterol biosynthesis and transportation, inflammation, and kidney function [163]. Although biomarkers from a shared pathway may indicate the mechanistic role and therapeutic potential of the pathway, intercorrelation with established risk factors can limit their contribution to the predictive value of a model already including those risk factors [163].

## 8. Systems Biology: Integrating Multidimensional Data

With advancement in technologies, the availability of multi-omics data such as sequencing data (gene and ribonucleic acid), proteomics, metabolomics, and lipidomics has made it possible to investigate diabetes and its complications using a systems biology approach [164]. A proportion of interindividual variability of metabolite concentrations can be explained by genetics. Variants identified in a large GWAS can account for up to 23% of the variance of metabolite concentrations [165]. Analysis performed in a Finnish Twin Cohort study found that the heritability estimates ranged between 23–55% for amino acids and other small-molecule metabolites and 48–76% for lipids and lipoproteins [166]. Some loci even explained up to 36% of the variance in circulating metabolites [167]. By using genetic variants associated with metabolites identified in GWAS as instrumental variables, MR can be utilized to make causal inferences with observational data. As genetic variants are randomly assigned during meiosis and fixed at conception, MR can overcome issues of residual confounding or reverse causality commonly observed in epidemiological studies [168]. If a metabolite is causally associated with diabetes or its complications, it may become possible to identify potential drugs targeting the underlying mechanism as a new treatment strategy. Moreover, the integration of multi-omics data or even clinical data using systems biology approaches may identify previously unappreciated inter-relationships between different biological or molecular pathways. For example, by combining metabolomics and transcriptomics via a metabolite–protein interaction network, four pathways associated with eGFR have been identified to be affected by dapagliflozin, which might shine a light on the potential renoprotective mechanisms of SGLT2i [116]. In contrast to the rapid development of “omics” technologies, statistical and computational techniques required to handle high-dimensional data, however, remain a major challenge and bottleneck [169].

## 9. Exogenous Metabolites, Gut Microbiota, and Diabetes and Its Progression

Exogenous inputs, such as food intake, affect the levels of circulating metabolites [170] and it has been increasingly appreciated that the gut microbiota play a key role in modifying the metabolome and metabolic homeostasis. Dietary phosphatidylcholines, including betaine, choline, and trimethylamine-N-oxide (TMAO), have been found to be altered in individuals with CVD and appear to promote development of atherosclerosis [171]. Higher plasma TMAO by LC-MS was also associated with CVE in patients with T2D [127]. A recent bidirectional two-sample MR found that genetically predicted TMAO was not associated with T2D, CKD, or CVD, whereas T2D and CKD were causally associated with higher TMAO, indicating that TMAO may play a mediating role between diabetes/CKD and CVD [172]. Using untargeted LC-MS, more microbial metabolites have been found to be predictive of incident diabetes in the METSIM study [63]. Studies integrating metabolomics with genetics and gut microbiota have been implemented to explore the interplay between genetic variants, dietary intake, gut microbiome and metabolites in diabetes and its complications [65,103].

## 10. Conclusions and Perspectives

Metabolomic studies present the molecular characterization of diabetes and its complications and could elucidate underlying pathological pathways that are perturbed in a disease state. Metabolomics, especially using the untargeted approach, can provide a global metabolic snapshot and may identify previously unknown molecules that are involved in the development and progression of diabetes. Metabolomic studies, as mentioned above, have identified biomarkers for the screening, diagnosis, and prediction of diabetes and its complications; some metabolites could also be biomarkers for monitoring the therapeutic effects of treatment. If being causal of a disease, the associated pathways could even be considered therapeutic targets. The integration of genetics, transcriptomics, proteomics, metabolomics, or even clinical data in a systems approach may present a comprehensive metabolic network of cardiometabolic disease. In this regard, metabolomics is a powerful approach which can deepen the molecular understanding of and improve efforts towards preventing or improving clinical management of T2D and its complications.

## Figures and Tables

**Table 1:** Advantages and disadvantages of different platforms for metabolomics studies.

	NMR	GC-MS	LC-MS
**Applications**	Targeted and untargeted	Targeted and untargeted	Targeted and untargeted
**Throughput**	10–30 min	20–60 min	15–60 min
**Advantages**	Nondestructive and suitable for in vivo	Sensitive	Highly sensitive
	Quantitative of absolute concentrations	Quantitative of absolute concentrations	Wide dynamic range
	Requiring little or no sample preparation	Robust and reproducible	No need for derivatization
	Automated and robust	Small sample volume required (~1 uL)	Small sample volume required (10–100 uL)
	Highly reproducible	Available databases for identification (i.e., NIST)	Compatible with solids and liquids
		Less expensive compared with LC-MS	
**Disadvantages**	Poor sensitivity	Destructive	Destructive
	Large sample volumes required (~0.5 mL)	Requiring derivatization and separation	Requiring separation
		Not compatible with solids	Lack of absolute quantification in untargeted applications
			Less reproducible
			Difficulty in unknown metabolite identification
			More expensive compared with GC-MS

GC-MS, gas chromatography–mass spectrometry; LC-MS, liquid chromatography–mass spectrometry; NIST, National Institute of Standards and Technology; NMR, nuclear magnetic resonance.

**Table 2:** Circulating metabolites associated with type 2 diabetes in prospective epidemiological studies.

Reference; Year	Study Design	Number, Follow-Up	Technique	Biological Matrix	Outcome	Adjustments	Major Findings	Replication
[36]; 2005	IRAS; America; population-based cohort	825 (129 T2D); 5.2 years	Targeted; NMR	Plasma	Incident T2D	Age, gender, and ethnicity	(+): VLDL particle, large VLDL, LDL particle, small LDL, small HDL, triglycerides; (−): large HDL, HDL cholesterol	No
[37]; 2010	WHS; America; randomized, double-blinded, placebo-controlled trial	26,836 (1687 T2D); 13.3 years	Targeted; NMR	Plasma	Incident T2D	Age, race, randomized treatment assignment, smoking, exercise, education, menopausal status, hormone use, blood pressure, BMI, family history of diabetes, HbA_1C_, and high-sensitivity C-Reactive protein	(+): total LDL particle, IDL particle, small LDL particle, small HDL particle, triglycerides, total VLDL particle, large VLDL particle, small VLDL particle (−): large LDL particle, HDL cholesterol, total HDL particle, large HDL particle	No
[38]; 2011	FHS; America; nested case–control	189 T2D and 189 control; 12 years	Targeted; LC-MS	Plasma	Incident T2D	Age, sex, BMI, FPG, and family history of T2D	(+): isoleucine, leucine, valine, tyrosine, phenylalanine	Yes; Malmö Diet and Cancer study, Sweden; nested case–control (163 T2D and 163 no T2D)
[39]; 2012	METSIM; Finland; population-based cohort	1775 (151 T2D); 4.7 years	Targeted; NMR	Serum	Incident T2D	Age, BMI	(+): alanine, isoleucine, leucine, phenylalanine, tyrosine; (−): glutamine	No
[40]; 2012	KORA; Germany; population-based cohort	589 (118 IGT) and 876 (91 T2D); 7 years	Targeted; LC-MS	Plasma	Incident IGT and T2D	Age, sex, BMI, physical activity, alcohol intake, smoking, systolic BP and HDL cholesterol	(−): glycine, LPC (18:2)	No
[41]; 2013	Botnia study; Finland; family-based study	2580 (151 T2D); 9.5 years	Targeted; LC-MS	Plasma	Incident T2D	Age, sex, BMI, family history of diabetes, and fasting glucose	(+): a-hydroxybutyrate; (−): L-GPC	No
[42]; 2013	EPIC; Germany; case–cohort	2282 (800 T2D); 7 years	Targeted; MS	Serum	Incident T2D	Age, sex, alcohol intake, smoking, physical activity, education, coffee intake, red meat intake, whole-grain bread intake, prevalent hypertension BMI, and waist circumference	(+): hexose, phenylalanine, diacyl-phosphatidylcholines (C32:1, C36:1, C38:3, C40:5); (−): glycine, sphingomyelin C16:1, lysophospha-tidylcholine C18:2, and acyl-alkyl-phosphatidylcholines (C34:3, C40:6, C42:5, C44:4, C44:5)	Yes; KORA, Germany; 876 (91 T2D); 7 years
[43], 2013	METSIM; Finland; population-based cohort	4306 (276 T2D); 5 years	Targeted; NMR	Plasma	Incident T2D	Age, BMI, smoking, and physical activity	(+): acetoacetate	No
[44]; 2013	METSIM; Finland; population-based cohort	4335 (276 T2D); 4.5 years	Targeted; NMR	Plasma	Incident T2D	Age, BMI, smoking, and physical activity	(+): glycerol, total fatty acids, triglycerides, monounsaturated fatty acids%, saturated fatty acids%; (−): *n*-3 fatty acids%, *n*-6 fatty acids%, linoleic acid%.	No
[45]; 2015	SABRE; Britain; population-based cohort	801 Europeans (113 T2D) and 643 South Asians (227 T2D); 19 years	Targeted; NMR	Serum	Incident T2D	Age, WHR, truncal skinfold thickness, Matsuda-IR, HDL-cholesterol level, current smoking, and alcohol consumption	(+): tyrosine for South Asians; (−): glycine for Europeans	No
[46]; 2015	METSIM; Finland; population-based cohort	6607 (386 T2D); 5.9 years	Targeted; NMR	Serum	Incident T2D	Age, BMI, smoking, and physical activity	(+): ApoA1/HDL-C ratio, ApoB/LDL-C ratio, ApoB/non-HDL-C ratio; (−): HDL cholesterol and large HDL particles	No
[47]; 2015	IRAS; America; population-based cohort	146 (76 T2D); 5 years	Targeted; MS/MS	Plasma	Incident T2D	Age, sex, BMI, and ethnicity	(+): alanine, valine, leucine or isoleucine, phenylalanine, glutamine and glutamate; (−): glycine, asparagine and aspartate	No
[48]; 2016	Four cohorts: ULSAM; Sweden, population-based cohort; PIVUS; Sweden, population-based cohort; the TwinGene study; Sweden, case–cohort; KORA; Germany, population-based cohort	1138 from ULSAM (78 T2D), 970 from PIVUS (70 T2D), 1630 from TwinGene (122 T2D), and 855 from KORA (88 T2D)	Untargeted; LC-MS	Plasma and serum	Incident T2D	Age, sex, BMI, waist circumference, and fasting glucose	(+): γ-glutamyl-leucine, 2-methylbutyroylcarnitine, barogenin, L-tyrosine, monoacylglycerol (18:2), deoxycholic acid; (−): cortisol, LysoPC/PC(O-16:1/0:0), SM (33:1, d18:2/18:1, 34:2), LysoPC (20:2), CerPE (38:2), PC (42:7)	No
[49]; 2016	Two Chinese cohorts: DFTJ and JSNCD; nested case–control studies	2078 from DFJT (1039 T2D); 4.6 years; 140 form JSNCD (520 T2D); 7.6 years	Targeted; LC-MS	Plasma	Incident T2D	Age, BMI, smoking and drinking status, education level, physical activity, systolic blood pressure, serum HDL cholesterol and triglycerides, fasting glucose, family history of diabetes, and metabolomics batch	(+): alanine, phenylalanine, tyrosine, palmitoylcarnitine	Yes
[50]; 2016	RISC; Europe, population-based cohort DMVhi; Britain; population-based cohort	855 (623 NGT, 56 isolated IGT (iIGT), 220 isolated IFG, 56 IFG and IGT); 3 years 668 (not given); 3 years	Targeted; LC-MS/MS	Plasma	iIGT	Age, sex, and BMI	(+): a-hydroxybutyric acid, oleic acid; (−): linoleoyl-glycerophosphocholine	Yes, Botnia, Finland; 2430 (not given)
[51]; 2016	SCHS; Singapore; nested case–control	394 (197 T2D); 6 years	Untargeted; LC-MS and GC-MS	Serum	Incident T2D	BMI, smoking status, and history of hypertension	(+): aminomalonic acid, glycine, isoleucine, leucine, threonine, valine, hippuric acid, cytidine diphosphate glucose, D-galactose, gluconate, palmitic acid (16:0), stearic acid (18:0), oleic acid (18:1), linoleic acid (18:2), LPG (12:0), LPI (16:1, 18:1, 18:2, 20:3, 20:4, 22:6), lactic acid, pyruvate, urea, 1,3-propanediol; (−): 2-aminooctanoic acid, ornithine, phosphoserine, proline, serine, glycerol, 9-decenoylcarnitine (C10), CMPF, LPE (20:3, 20:5)	No
[52]; 2017	Botnia Prospective Study; Finland; population-based cohort	543 (146 T2D); 7.7 years	Untargeted and targeted; MS	Serum	Incident T2D	Age, sex, BMI, fasting insulin level, and family history of type 2 diabetes	(+): glucose, mannose, α-hydroxybutyrate, isoleucine, valine, glutamate, trehalose; (−): histidine, bilirubin, glutamine, α-Tocopherol	Yes; DESIR, France; 1044 (231 T2D); 9 years
[53]; 2017	ERF; Netherlands; population-based cohort	1571 (137 T2D); 11.3 years	Targeted; NMR and LC-MS	Plasma	Incident T2D	Age, sex, and lipid-lowering medication	(+): isoleucine, tyrosine, 2-hydroxybutyrate, 2-oxoglutaric acid, glycerol, lactate, pyruvate, TG (48:0), TG (48:1), TG (50:5), VLDL free cholesterol, extremely large VLDL cholesterol, VLDL triglycerides, very small LDL and ApoB	No
[54]; 2018	The Västerbotten Intervention Programme cohort; Sweden; nested case–control study	1006 (503 T2D); 7 years	Untargeted; LC-MS	Plasma	Incident T2D	BMI and FPG	(+): PC(16:0/16:1), DAG(16:1/16:1, 14:0/16:0, 14:0/18:1, 16:0/18:1), 3-hydroxyisovalerylcarnitine, phenylalanine, leucine, isoleucine, valine, tryptophan, L-tyrosine, alanine, citrulline; (−): lysoPC (18:2, 18:1, p16:0, 17:0, 19:1, 20:1), PC (15:1/18:2, 17:0/18:2), n-acetylglycine, 2-hydroxyethanesulfonate, 3-methyl-2-oxovaleric acid	No
[55]; 2018	SCHS; Singapore; nested case–control study	320 (160 T2D); not given	Targeted; LC-MS and GC-MS	Serum	Incident T2D	BMI, history of hypertension, smoking, physical activity, fasting status, triglycerides, and HDL-cholesterol	(+): lysophosphatidylinositol (16:1, 18:0), myristic acid (14:0), palmitic acid (16:0), palmitoleic acid (16:1n-7), stearic acid (18:0), eicosadienoic acid (20:2n-6), dihomo-gamma-linolenic acid (20:3n-6), arachidonic acid (20:4n-6), adrenic acid (22:4n-6)	No
[56]; 2018	KoGES; community-based cohort	1939 (282 T2D); 8 years	Targeted; MS	Serum	Incident T2D	Sex, age, energy intake, body-mass index, metabolic equivalent, smoking status, drinking status, household income, and education level, consumption of coffee, red meat, and whole grain, and history of hypertension	(+): alanine, arginine, isoluecine, proline, tyrosine, valine, hexose, phosphatidylcholine diacyl (C32:1, C34:1, C36:1, C40:5, C42:5); (−): spermine, lyso phosphatidylcholine acyl (C17:0, C18:2, C38:0, C40:1, C42:1, C34:3, C36:3), hydroxysphingomyelin C22:2, sphingomyelin C16:1	No
[57]; 2018	ARIC; America; community-based cohort	2939 (1126 T2D); 20 years	Untargeted; LC-MS	Serum	Incident T2D	Age, sex, race, center, batch, education level, systolic blood pressure, diastolic blood pressure, BMI, HDL-cholesterol, LDL-cholesterol, smoking status, physical activity level, history of cardiovascular disease, eGFR, and fasting glucose	(+): isoleucine, leucine, 3-(4-hydroxyphenyl)lactate, valine, trehalose, erythritol; (−): asparagine	No
[58]; 2018	FHS; America; community-based cohort	1150 with NFG (95 T2D); 20 years	Targeted; LC-MS/MS	Plasma	Incident T2D	Age, sex, BMI, fasting glucose, and triglycerides	(+): phenylalanine; (−): glycine, taurine	No
[59]; 2019	Four Finnish population-based cohorts: YFS; FINRISK-1997; DILGOM; NFBC	11,896 (392 T2D); 8–15 years	Targeted; NMR	Serum	Incident T2D	Sex, baseline age, BMI, and fasting glucose	(+): isoleucine, leucine, phenylalanine, glycerol, glycoprotein acetyls, total fatty acids, monounsaturated fatty acids%, triacylglycer/phosphoglyceride ratio, VLDL cholesterol, total triacylglycerol, triacylglycerol in VLDL, triacylglycerol in LDL, apo B/apo A1 ratio, VLDL particle size; (−): polyunsaturated fatty acids%, *n*-6 fatty acids%, linoleic acid%, HDL cholesterol, HDL particle size	No
[60]; 2019	METSIM; Finland; population-based cohort	4851 (522 T2D); 7.4 years	Untargeted; LC-MS	Plasma	Incident T2D	Batch effect, age, BMI, smoking, and physical activity	(+): tyrosine, alanine, isoleucine, aspartate, glutamate	No
[61]; 2019	MPP; Sweden; case–cohort study	698 (202 T2D); 6.3 years	Untargeted; LC-MS	Plasma	Incident T2D	Age, sex, fasting glucose, and BMI	(+): N2,N2-dimethylguanosine, 7-methylguanine, 3-hydroxy-trimethyllysine, urea	Yes, MDC-CC, Sweden; population-based cohort; 3423 (402 T2D); 18.2 years
[62]; 2019	PREDIMED; Spain; case–cohort	853 (243 T2D); 3.8 years	Targeted; LC-MS	Plasma	Incident T2D	Age, sex, intervention, BMI, smoking, dyslipidemia, hypertension, and baseline plasma glucose	(+): lysine, 2-aminoadipic acid	No
[63]; 2020	METSIM; Finland; population-based cohort	4851 (522 T2D); 7.4 years	Untargeted; LC-MS	Plasma	Incident T2D	Age, BMI, smoking, and physical activity	(+): creatine; 1-palmitoleoylglycerol (16:1), urate, 2-hydroxybutyrate, xanthine, xanthurenate, kynurenate, 3-(4-hydroxyphenyl) lactate, 1-oleoylglycerol (18:1), 1-myristoylglycerol (14:0), dimethylglycine, 2-hydroxyhippurate; (−): 1-linoleoyl-GPC (18:2)	No
[64]; 2021	DFTJ; China; nested case–control	1000 (500 T2D); 4.61 years	Untargeted; LC-MS	Serum	Incident T2D	Age, sex, BMI, smoking status, drinking status, and physical activity	(+): carnitine 14:0, PE 34:2, FFA 20:4; (−): pipecolinic acid, 1,5-Anhydro-D-Glucitol, LPC 18:2, Isoleucine/leucine, epinephrine	No
[65]; 2021	Five cohorts from America: HCHS/SOL; ARIC; FHS, WHI and a case–cohort study nested in PREDIMED; prospective	9180 (2031 T2D); 5.7 years	LC-MS	Serum and plasma	Incident T2D	Age, sex, smoking, alcohol consumption, education, family income, family history of diabetes, self-reported hypertension and/or antihypertensive medication use, self-reported dyslipidemia and/or lipid-lowering medication use, other study-specific covariates, BMI and WHR; yes	(+): tryptophan, kynurenine, kynurenate, xanthurenate, quinolinate; (−): indolepropionate	No
[66]; 2021	PREVEND; Netherlands; population-based cohort	4828 (265 T2D); 7.3 years	Targeted; NMR	Plasma	Incident T2D	Age, sex, family history of diabetes, smoking, alcohol assumption, BMI, hypertension, high-sensitivity C-reactive protein, lipid-lowering medication, and fasting glucose	(+): small HDL; (−): HDL cholesterol, large HDL, medium HDL	No

IRAS, Insulin Resistance Atherosclerosis Study; WHS, Women’s Health Study; FHS, Framingham Heart Study; METSIM, Metabolic Syndrome in Men; KORA, Cooperative Health Research in the Region of Augsburg; EPIC, European Prospective Investigation into Cancer and Nutrition; SABRE, Southall Additionally, Brent Revisited; ULSAM, Uppsala Longitudinal Study of Adult Men; PIVUS, Prospective Investigation of the Vasculature in Uppsala Seniors; DFTJ, Dongfeng-Tongji; JSNCD, Jiangsu Noncommunicable Disease; RISC, Relationship Between Insulin Sensitivity and Cardiovascular Disease; SCHS, Singapore Chinese Health Study; DESIR, Data from an Epidemiological Study on the Insulin Resistance Syndrome; ERF, Erasmus Rucphen Family genetic isolate study; KoGES, Korean Genome and Epidemiology Study; ARIC, Atherosclerosis Risk in Communities; YFS, Cardiovascular Risk in Young Finns Study; DILGOM, Dietary, Lifestyle, and Genetic Determinants of Obesity and Metabolic Syndrome; NFBC, Northern Finland Birth Cohort; MPP, Malmö Preventive Project; MDC-CC: Malmö Diet and Cancer–Cardiovascular Cohort; PREDIMED, Prevención con Dieta Mediterránea study; HCHS/SOL, Hispanic Community Health Study/Study of Latinos; PREVEND: Prevention of Renal and Vascular End-Stage Disease; BMI, body-mass index; CerPE, ceramide phosphoethanolamine; CMPF, 3-carboxy-4-methyl-5-propyl-2-furanpropionic acid; DAG, diglyceride; eGFR, estimated glomerular filtration rate; FFA, free fatty acid; FPG, fasting plasma glucose; GPC, glycerophosphocholine; HbA_1C_, glycated hemoglobin; IGT, impaired glucose tolerance; IR, insulin resistance; L-GPC, linoleoyl-glycerophosphocholine; LPC, lysophosphatidylcholine; LPE, lysophosphatidylethanolamine; LPG, lysophosphatidylglycerol; lysoPC, lysophosphatidylcholine; NFG, normal fasting glucose; NGT, normal glucose tolerance; PC, phosphatidylcholine; PE, phosphatidylethanolamine; PI, lysophosphatidylinositol; SM, sphingomyelin; T2D, type 2 diabetes; TG, triacylglycerol; WHR, waist-hip ratio.

**Table 3:** Circulating metabolites associated with diabetic kidney disease in human studies.

Reference; Year	Study Design	Number, Follow-Up	Technique	Biological Matrix	Outcome, Number	Adjustments	Major Findings	Replication
[81]; 2009	China; case–control	119 (31 control: no DM and DN, 23 T2D without DN, 65 T2D and DN)	Targeted; LC-MS	Plasma	NA	NA	Higher levels of inosine, adenosine, uric acid, and xanthine in DN group compared with control or T2D without DN group	No
[82]; 2012	Japan; case–control	78 T2D (20 normoalbuminuria, 32 microalbuminuria, 26 macroalbuminuria)	Untargeted; MS	Serum	NA	No	Higher levels of creatinine, aspartic acid, γ-butyrobetaine, citrulline, SDMA and kynurenine and lower levels of azelaic acid and galactaric acid in macroalbuminuria group compared with normoalbuminuria group	No
[83]; 2012	FinnDiane; Finland; nested case–control	52 T1D (26 progressing to micro/macroalbuminuria, 26 nonprogressor); 5.5 years	Untargeted; LC-MS and GC-MS	Urine	Progression from normoalbuminuria to micro- or macro-albuminuria; 26	No	Higher level of substituted carnitine and S-(3-oxododecanoyl) cysteamine and lower level of hippuric acid in progressors	No
[84]; 2012	FinnDiane; Finland; cross-sectional	326 T1D (182 normal AER, 58 microalbuminuria, 86 macroalbuminuria)	Targeted; NMR	Serum	24 h AER	Diabetes duration, gender, waist, SBP, HbA_1C_, triglycerides, HDL cholesterol, and serum creatinine	(+): sphingomyelin	No
[85]; 2013	America; case–control	47: 23 healthy control, 24 T2D with CKD (screening group)	Targeted; GC-MS	Urine and plasma	NA	Age, race, and sex	Lower levels of urine 3-hydroxy isovalerate, aconitic acid, citric acid, 2-ethyl 3-OH propionate, glycolic acid, homovanillic acid, 3-hydroxyisobutyrate, 2-methylacetoacetate, 3-methyladipic acid, 3-methylcrotonylglycine, 3-hydroxypropionate, tiglylglycine, and uracil in DM with CKD group compared with control group	Yes; 61 diabetes (12 T1D and 49 T2D) with CKD as validation group
[86]; 2014	PREVEND; Netherlands; The Steno Diabetes Center; Denmark; nested case–control	90 T2D (24 from normoalbuminuria to microalbuminuria, 24 normoalbuminuria control; 21 from microalbuminuria to macroalbuminuria, 21 microalbuminuria control); 2.9 years	Targeted; LC-MS	Urine and Plasma	Transition from normo- to micro-albuminuria or from micro- to macro-albuminuria; 24 from normo- to micro-albuminuria, 21 from micro- to macro-albuminuria	Baseline UAE and eGFR	Higher plasma levels of butenoylcarnitine and lower levels of plasma histidine, urine hexose, urine glutamine, and urine tyrosine in patients progressing from microalbuminuria to macroalbuminuria compared with controls	No
[87]; 2014	DCCT; America; prospective	497 T1D; 14–20 years	Targeted; LC-MS	Plasma	Incident macroalbuminuria; 65	DCCT Treatment Group, baseline retinopathy status, use of ACE/ARB drugs during study period, gender, and baseline measures of duration of T1DM, age, HbA_1C_ %, BMI, triglyceride levels, and AER	(−): very long chain ceramide species (C20, C22:1, C24, C26, and C26:1)	No
[88]; 2014	The Joslin Study of the Genetics of Type 2 Diabetes and Kidney Complications; America; nested case–control	80 T2D (40 incident ESRD, 40 without ESRD); 8–12 years	Targeted and untargeted; LC-MS and GC-MS	Plasma	Incident ESRD: renal death, renal replacement therapy	HbA_1C_, AER, and eGFR	(+): p-cresol sulfate, gulono-1,4-lactone, threitol, erythronate, pseudouridine, N2,N2-dimethylguanosine, N4-acetylcytidine, C-glycosyltryptophan, glutaroyl carnitine, methylglutarylcarnitine, 3-dehygrocarnitine, urea, myo-inositol, urate, phenylacetylglutamine; (−): 2-hydroxyisocaproate, 2-oxoisoleucine, 2-hydroxyisovalerate, 2-hydroxybutyrate	No
[89]; 2015	GO-DARTS; Scotland; nested case–control	307 T2D with baseline eGFR 30–60 mL/min/1.73 m^2^; 3.5 years	Targeted; LC-MS	Serum	Rapid eGFR progression: >40% compared with baseline; 154	Age, sex, eGFR, albuminuria status, HbA_1C_, use of ACE inhibitors, and use of ARBs	(+): C16-acylcarnitine, creatinine, methylmalonic acid, n-acetylaspartate, sialic acid, SDMA, SDMA/ADMA, uracil; (-): lysine, tryptophan	No
[90]; 2016	Singapore; case–control	129 T2D without DKD (control), 126 T2D with ACR 30–299 mg/g and eGFR 60 mL/min/1.73 m^2^ (early DKD), 154 T2D with ACR ≥300 mg/g or eGFR <60 mL/min/1.73 m^2^ (overt DKD)	Targeted; LC-MS and GC-MS	Plasma	NA	Age, sex, and ethnicity	Higher levels of C2, C3, C4, C4-OH, C5, C4-DC, C5:1, C5-DC, C5-OH/C3-DC, C6, C8-OH/C6-DC, C14:1-OH, C14-OH/C12-DC, C18-OH/C16-DC acylcarnitines, Cer 18:1/16:0, GlcCer 18:1/18:0, SM 18:1/16:1, and sphingosine and lower levels of serine, (32:2, 34:3, 36:6, 38:3, 40:5) in overt DKD compared with control group	Yes, 149 T2D without DKD, 149 T2D with overt DKD
[91]; 2016	Italy; prospective	286 T2D; 3 years	Untargeted; LC-MS and GC-MS	Urine and serum	Association with baseline eGFR and ACR; incident >10 mL/min/1.73 m^2^ eGFR decline; incident >14 mg/g ACR increase; number not given	Gender, age, glucose, and baseline eGFR	(+): C-glycosyl tryptophan, pseudouridine, N-acetylthreonine	No
[92]; 2017	China; case–control	20 healthy controls (control); 25 T2D with UACR <30 mg/g (T2D); 24 T2D with UACR ≥30 mg/g (DKD)	Untargeted; GC-MS	Urine	NA	No	Higher levels of uric acid, stearic acid, palmitic acid, and hippuric acid and lower levels of uracil, glycine, aconitic acid, isocitric acid, 4-hydroxybutyrate, glycolic acid, and 2-deoxyerythritol in DKD compared with control or compared with T2D group	No
[93]; 2017	The Joslin Proteinuria Cohort Study; America; prospective	158 T1D with proteinuria and stage three CKD; 11 years	Targeted; LC-MS and GC-MS	Serum	Incident ESRD: renal death or renal replacement therapy; 99	Blood pressure, BMI, smoking status, HbA_1C_, ACR, eGFR, uric acid levels, treatment with renin-angiotensin system inhibitors, other antihypertensive treatment, and statins	(+): n-acetylserine, n-acetylthreonine, n6-acetyllysine, n6-carbamoylthreonyladenosine, c-glycosyltryptophan, pseudouridine, o-sulfotyrosine	No
[94]; 2018	FinnDiane; Finland; nested case–control	200 T1D (102 progressing to microalbuminuria, 98 nonprogressors); 3.2 and 7.1 years, respectively	Untargeted; LC-MS and GC-MS	Serum	Progression to microalbuminuria; 102	Age of diabetes onset, HbA_1C_, and AER	(+): erythritol, 3-phenylpropionate, N-trimethyl-5-aminovalerate	No
[95]; 2018	ADVANCE; Australia; case–cohort	3587 T2D; 5 years	Targeted; NMR	Plasma	Major microvascular events: a composite of new or worsening nephropathy or retinopathy; 342	Age, sex, region and randomized treatment, a prior macrovascular complication, duration of diabetes, current smoking, systolic blood pressure, BMI, ACR, eGFR, HbA_1C_, plasma glucose, total cholesterol, HDL-cholesterol, triacylglycerols, aspirin or other antiplatelet agent, statin or other lipid-lowering agent, β-blocker, ACE inhibitor or angiotensin receptor blocker, metformin use, history of heart failure, participation in moderate and/or vigorous exercise for >15 min at least once weekly, and high-sensitivity CRP	(−): alanine, tyrosine	No
[96]; 2018	Macroalbuminuric DKD; Brazil; prospective	56 with T2D; 2.5 years	Untargeted, GC-MS	Plasma	All-cause death, doubling of baseline serum creatinine and/or dialysis initiation; 17	eGFR	(−): 1,5-anhydroglucitol, norvaline, l-aspartic acid	No
[97]; 2018	GenodiabMar; not given; TwinsUK; Britain; KORA; Germany; prospective	655 T2D from GenodiabMar; 111 T2D from TwinsUK; 160 T2D from KORA; cross-sectional	Targeted; NMR	Serum	Association with baseline eGFR; 926	Age, gender, and BMI	(+): apolipoprotein A1, total cholesterol in HDL2, total cholesterol in very large HDL, total cholesterol in HDL, free cholesterol in medium HDL, cholesterol esters in very large HDL, concentration of very large HDL particles, concentration of medium HDL particles, total lipids in medium HDL, phospholipids in medium HDL, esterified cholesterol, total cholesterol, total cholesterol in large LDL, total cholesterol in large LDL, total cholesterol in medium LDL, total cholesterol in small LDL, total cholesterol in LDL, total cholesterol in IDL, free cholesterol in large LDL, free cholesterol in medium LDL, free cholesterol in small LDL, free cholesterol in IDL, cholesterol esters in large LDL, cholesterol esters in medium LDL, cholesterol esters in small LDL, cholesterol esters in IDL, concentration of large LDL particles, concentration of IDL particles, total lipids in large LDL, total lipids in medium LDL, total lipids in small LDL, total lipids in IDL, phospholipids in large LDL, phospholipids in medium LDL, phospholipids in small LDL, phospholipids in IDL; (−): glycine, phenylalanine, citrate, glycerol	No
[98]; 2019	The Renoprotection in Early Diabetic Nephropathy in Pima Indians trial; America; prospective	92 T2D with baseline eGFR ≥90 mL/min/1.73 m^2^; 9.6 years	Untargeted; LC-MS	Serum	≥40% reduction in eGFR compared with baseline; 32	GFR and ACR	(+): unsaturated PEs; (−): unsaturated FFAs	No
[99]; 2019	Denmark; prospective cohort study	637 T1D; 5.5 years	Targeted; GC-MS	Serum	Combined renal endpoint: ≥30% decrease in eGFR, ESRD, or all-cause mortality; 123	Age, sex, HbA_1C_, SBP, smoking, BMI, statin treatment, triglycerides, total cholesterol, eGFR, and logAER	(+): ribonic acid; (−): isoleucine, leucine, valine	No
[100]; 2019	China; nested case–control	52 T2D with macroalbuminuria and eGFR >90 mL/min/1.73 m^2^ (25 progressors and 27 nonprogressors); 5–6 years	Targeted and untargeted; LC-MS	Urine	Early progressive renal function decline: at least a 33.3% decline of eGFR and eGFR <60 mL/min/1.73 m^2^; 25	No	(−): 5-hydroxyhexanoic acid	No
[101]; 2019	GoDARTS; Scotland; nested case–control; SDR; Sweden; prospective; CARDS; Britain; clinical trial	430 T2D from GoDARTS, 227 T2D from SDR, 183 from CARDS; 7 years	MS	Serum	>20% eGFR reduction compared with baseline; 403	Age, sex, baseline eGFR, albuminuria, HbA_1C_, and calendar time	(+): ADMA, SDMA	No
[102]; 2019	SDRNT1BIO; Scotland; prospective	859 T1D with baseline eGFR 30–75 mL/min/1.73 m^2^; 5.2 years	Targeted; LC-MS	Serum	Rapid eGFR decline during follow-up: > 3 mL/min/1.72 m^2^/year; 194	Age, sex, duration of diabetes, study day eGFR, and length of follow-up	(+): free sialic acid; (−): tryptophan/kynurenine, threonine, methionine, tryptophan	No
[103]; 2020	Denmark; case–control	211 (50 heathy control, 161 T1D: 50 normoalbuminuria, 50 micoralbuminuria, 61 macroalbuminuria); cross-sectional	Targeted; MS	Plasma	NA	Use of medication, HbA_1C_, and diabetes duration	Higher levels of indoxyl sulphate, L-citrulline in T1D and macroalbuminuria group compared with normo-or microalbuminuria group; higher levels of homocitrulline, L-kynurenine and lower level of tryptophan in macroalbuminuria group compared with normoalbuminuria group	No
[104]; 2020	KORA; Germany; population-based cohort	385 prediabetes or T2D; 6.5 years	Targeted; LC-MS	Serum	Incident CKD: eGFR <60 mL/mL/min/1.73 m^2^ and/or UACR ≥ 30 mg/g; 85	Age, sex, BMI, SBP, smoking status, triglyceride, total cholesterol, HDL cholesterol, fasting glucose, use of lipid-lowering, antihypertensive and antidiabetic medications, baseline eGFR, and ACR	(+): PC aa (C38:0, C42:0, C40:6), SM (OH) (C14:1, C16:1), SM (C16:0, C16:1, C18:0, C18:1, C20:2, C24:1, C26:1); (−): PC aa C32:2	No
[105]; 2020	CRIC; America; prospective cohort study	1001 diabetes with baseline eGFR 20–70 mL/min/1.73 m^2^; 8 years	Untargeted; MS	Urine	ESRD (incident kidney failure with replacement therapy); 359	Age, race, sex, smoked more than 100 cigarettes, BMI, HbA_1C_, mean arterial pressure, AER, and baseline eGFR	(+): 3-hydroxypropionate, 3-hydroxyisobutyrate, glycolic acid	No
[106]; 2020	5 Dutch cohort studies: DCS West-Friesland, the Maastricht study, the Rotterdam study, the Netherlands Epidemiology of Obesity study, the Cohort of Diabetes and Atherosclerosis Maastricht study	3089 T2D; 4–7 years	Targeted; NMR	Plasma	Cross-sectional association with baseline eGFR and ACR	Age, sex, use of statins, other lipid-modifying agents, oral glucose-lowering medications, insulins, RAS-blocking agents and other antihypertensives, SBP, BMI, smoking, diabetes duration, HbA_1C_, and baseline ACR/UAE	1) For baseline eGFR: (+): tyrosine, lactate, glucose, HDL particle, HDL cholesterol, apo A1, (−): phenylalanine, isoleucine, glutamine, histidine, leucine, glycoprotein acetyls, citrate, acetoacetate, VLDL particle, non-HDL cholesterol, triglycerides, lipid components in IDL and LDL 2) for baseline ACR: (+): glucose, glycoprotein acetyls, phosphatidylcholine and other cholines, free cholesterol in large VLDL; (−): very large HDL particle, glutamine	No
[107]; 2020	FinnDiane; Finland; nationwide prospective cohort	1087 T1D; 10.7 years	Targeted; NMR	Serum	Fastest eGFR decline: highest quartile of eGFR decline over follow up (−4.4 mL/min/1.73 m^2^) and progression from macroalbuminuria to ESRD	Age at diabetes onset, sex, diabetes duration, smoking, SBP, HbA_1C_, BMI, HDL cholesterol, and triacylglycerols	(+): sphingomyelin	No

FinnDiane, Finnish Diabetic Nephropathy Study Group; PREVEND, Prevention of Renal and Vascular End-stage Disease; DCCT, Diabetes Control and Complications Trial; GO-DARTS, Genetics of Diabetes Audit and Research Tayside Study; ADVANCE, Action in Diabetes and Vascular Disease: Preterax and Diamicron Modified Release Controlled Evaluation; SDR, Scania Diabetes Registry; CARDS, Collaborative Atorvastatin in Diabetes Study; SDRNT1BIO, Scottish Diabetes Research Network Type 1 Bioresource; CRIC, The Chronic Renal Insufficiency Cohort; DCS, Hoorn Diabetes Care System; ACE, angiotensin converting enzyme; ADMA, asymmetric dimethylarginine; AER, albumin excretion rate; Apo A1, apolipoprotein A1; ARB, angiotensin receptor blocker; Cer, ceramide; CRP, C-reactive protein; DN, diabetic nephropathy; FFAs, free fatty acids; GlcCer, glucosylceramide; PC; phosphatidylcholine; Pes, phosphatidylethanolamines; SDMA, symmetric dimethylarginine; SM, sphingomyelin; UAE, urinary albumin excretion.

**Table 4:** Circulating metabolites associated with cardiovascular disease in individuals with diabetes.

Reference; Year	Study Design	Number, Follow-Up	Technique	Biological Matrix	Outcome, Number	Adjustments	Major Findings	Replication
[121]; 2003	EDC; America; nested case–control	118 T1D (59 coronary artery disease); 10 years	Targeted; NMR	Plasma	Fatal or nonfatal myocardial infarction, angina, coronary stenosis >50%; 59	eGDR, smoking, overt nephropathy, retinopathy, WHR, and blood-pressure lowering drugs	(+): medium HDL particle, VLDL particle (−): large HDL particle	No
[122]; 2006	Austria; cross-sectional	136 T2D	Targeted; LC	Plasma	Macrovascular disease: history of stroke, myocardial infarction, coronary heart disease or peripheral arterial occlusive disease; 55	L-arginine, AER, homocysteine, and eGFR	(+): ADMA	No
[123]; 2007	SDC; Denmark; prospective	572 T1D (397 with overt DN, 175 with persistent normoalbuminuria); 11.3 years	Targeted; LC	Plasma	fatal and nonfatal cardiovascular disease; 116	Sex, age, HbA_1C_, SBP, GFR, cholesterol, smoking status, previous CVD events, antihypertensive treatment, NT-proBNP, and CRP	(+): ADMA	No
[124]; 2007	Austria; prospective	125 T2D; 21 months	Targeted; LC	Plasma	Cardiovascular events: myocardial infarction, percutaneous coronary intervention, coronary artery bypass graft, stroke, carotid revascularization, and all-cause mortality; 48	Age, sex, history of macrovascular disease, and GFR	(+): ADMA	No
[125]; 2014	The Shiga Prospective Observational Follow-up Study; Japan; prospective	385 T2D; 10 years	Targeted; LC-MS	Plasma	Cardiovascular composite endpoints: myocardial infarction, angina pectoris, worsening of congestive heart failure, and stroke; 63	Age, SBP, hypertension, log (HDL cholesterol), log (AER), eGFR, and baPWV	(+): cardiovascular disease-amino acid-based index composed of ethanolamine, hydroxyproline, glutamic acid, 3-methylhistidine, tyrosine, tryptophan	No
[126]; 2016	China; case–control	15 healthy control, 13 CHD, 15 T2D, 28 T2D and CHD	Untargeted; NMR	Plasma		No	Higher levels of VLDL/LDL, glucose and lower levels of isoleucine, valine, isopropanol, alanine, leucine, arginine, acetate, proline, glutamine, creatine, creatinine, glycine, threonine, tyrosine, 3-methylhistidine in T2D and CHD compared with healthy control	No
[95]; 2018	ADVANCE; Australia; case–cohort	3587 T2D; 5 years	Targeted; NMR	Plasma	Macrovascular events: cardiovascular death, nonfatal myocardial infarction or nonfatal stroke; 655	Age, sex, region and randomized treatment, a prior macrovascular complication, duration of diabetes, current smoking, systolic blood pressure, BMI, ACR, eGFR, HbA_1C_, plasma glucose, total cholesterol, HDL-cholesterol, triacylglycerol, aspirin or other antiplatelet agent, statin or other lipid-lowering agent, β-blocker, ACE inhibitor or angiotensin receptor blocker, metformin use, history of heart failure, participation in moderate and/or vigorous exercise for >15 min at least once weekly, and high-sensitivity CRP	(+): phenylalanine before fully adjustment (−): glutamine, histidine before full adjustment	No
[107]; 2020	FinnDiane; Finland; nationwide prospective cohort	1087 T1D; 10.7 years	Targeted; NMR	Serum	Coronary heart disease: myocardial infarction or coronary revascularisation; 110	Age at diabetes onset, sex, diabetes duration, and smoking	(+): sphingomyelin	No
[127]; 2020	SURDIAGENE; France; prospective	1463 T2D; 85 months	Targeted; LC-MS	Plasma	Major adverse cardiovascular events: a composite of CV death, nonfatal MI, nonfatal stroke; 403	Sex, age, MI history, eGFR, ACR, and NT-proBNP	(+): TMAO	No
[112]; 2020	ADVANCE; Australia; case–cohort	3576 T2D; 5 years	Targeted; NMR	Plasma	Major macrovascular events: cardiovascular death, fatal myocardial infarction and nonfatal stroke; 654	Age, sex, region and the treatments randomly allocated, history of macrovascular disease, duration of diabetes, current smoking status, SBP, BMI, ACR, eGFR, HbA_1C_, HDL-cholesterol, triacylglycerol, and use of aspirin or other antiplatelet agents, statins or other lipid-lowering agents, β-blockers and ACE inhibitors or angiotensin receptor blockers	(−): *n*-3 fatty acids, DHA	No

EDC, Pittsburgh Epidemiology of Diabetes Complications; SDC, Steno Diabetes Center; SURDIAGENE, SURVIe, DiAbete de type 2 et GENEtique; baPWV, brachial-ankle pulse wave velocity; DHA, docosahexaenoic acid; eGDR, estimated glucose disposal rate; NT-proBNP, N-terminal pro b-type natriuretic peptide; TMAO, rimethylamine N-oxide.

## Data Availability

Not applicable.
